# Nutrition of marine mesograzers: integrating feeding behavior, nutrient intake and performance of an herbivorous amphipod

**DOI:** 10.7717/peerj.5929

**Published:** 2018-11-09

**Authors:** Glauco B.O. Machado, Fosca P.P. Leite, Erik E. Sotka

**Affiliations:** 1 Programa de Pós-graduação em Ecologia, Instituto de Biologia, Universidade Estadual de Campinas, Campinas, São Paulo, Brazil; 2 Departamento de Biologia Animal, Instituto de Biologia, Universidade Estadual de Campinas, Campinas, São Paulo, Brazil; 3 Grice Marine Laboratory and the Department of Biology, College of Charleston, Charleston, SC, United States of America

**Keywords:** Geometric framework, Nutritional ecology, Ampithoidae, Feeding preference, Fitness, Seaweed-herbivore interaction

## Abstract

Consumers can regulate the acquisition and use of nutrients through behavioral and physiological mechanisms. Here, we present an experimental approach that simultaneously integrates multiple nutritional traits, feeding assays, and juvenile performance to assess whether a marine herbivore (the amphipod *Ampithoe valida*) regulates the intake of elements (carbon and nitrogen), macronutrients (protein and non-protein) or both when offered freeze-dried tissues of seaweeds varying in nutritional content. We assessed behavioral regulation of nutrients in three ways. First, during no-choice assays, we found that amphipods ingested similar amounts of carbon, but not nitrogen, non-protein and protein, across algal diets. Second, herbivore intake rates of carbon, protein and non-protein components across no-choice assays was similar to intake rates when offered a choice of foods. Third, variation in intake rates of carbon and non-protein components among algal diets was significantly greater than was tissue content of these components, while variation in intake rates of nitrogen was significantly lower; differences in protein intake variation was equivocal. While these analytical approaches are not uniformly consistent, carbon and nitrogen seem to emerge as the nutrient components that are more strongly regulated by *A. valida*. Juveniles reared on single diets shown patterns of survivorship, growth and reproduction that could not be predicted by these feeding preferences, nor nutrient content. We conclude that an integrative approach that considers the intake of multiple nutrients potentially yields insights into feeding behavior and its performance consequences.

## Introduction

Fulfilling nutritional requirements is challenging and affects how organisms interact with their biotic and abiotic environment (Raubenheimer et al., 2005; Hawlena & Schmitz, 2010; Rothman, Raubenheimer & Chapman, 2011; Simpson & Raubenheimer, 2011). One approach to understanding the intake and use of nutrients by consumers in the face of such ecological complexity is the geometric framework of nutrition (Raubenheimer & Simpson, 1993; Simpson & Raubenheimer, 1995), which has been extensively applied to a variety of consumer species (Lee et al., 2002; Raubenheimer & Simpson, 2003; Raubenheimer et al., 2005; Felton et al., 2009; Hawlena & Schmitz, 2010; Rothman, Raubenheimer & Chapman, 2011; Heflin et al., 2016). By integrating nutrient intake with behavioral and performance responses of consumers, the geometric framework allows investigators to explore the adaptive value of behavioral and physiological mechanisms involved in the ingestion and regulation of multiple nutrients (Raubenheimer, Simpson & Mayntz, 2009; Simpson & Raubenheimer, 2011).

Generalist herbivores often deal with nutritionally-imbalanced set of plants in their environment (Felton et al., 2009; Rothman, Raubenheimer & Chapman, 2011) by making feeding decisions (Zanotto, Simpson & Raubenheimer, 1993; Lee et al., 2002; Raubenheimer et al., 2005; Heflin et al., 2016). Herbivores that mix their diet can obtain a required balance of nutrients (Raubenheimer & Simpson, 1993; Raubenheimer & Simpson, 2003). However, when forced to feed on single foods by ecological factors, herbivores are constrained to ingest a specific ratio of nutrients, regardless the absolute amount of food consumed. If this ratio of nutrients does not match the nutritional balance required, consumers either compensate the low content of some nutrients by over-ingesting others, or under-ingest some nutrients to avoid ingesting in excess other food components (Raubenheimer & Simpson, 1993; Zanotto, Simpson & Raubenheimer, 1993; Raubenheimer & Simpson, 2003).

Insight into these decisions has been accelerated by the geometric framework of nutrition, in which the intake of two nutrients by a consumer fed on single diets varying in ratio of nutrients is graphically shown, with each axis representing a nutrient (e.g.,  Lee et al., 2002; Raubenheimer et al., 2005; Hawlena & Schmitz, 2010). The shape of the array resulting from nutrient intake observed for each diet can reveal the decision rules applied by a consumer when it deals with imbalanced foods (Raubenheimer & Simpson, 1993; Simpson & Raubenheimer, 1995). For example, considering two nutrients, if only the intake of one nutrient is regulated by the consumer, we could expect to observe similar intake of that nutrient across all diets regardless the intake of the other nutrient (i.e., all points are orthogonally arranged to the axis of the nutrient that is regulated) (e.g.,  Trumper & Simpson, 1993; Raubenheimer et al., 2005; Harrison et al., 2014). Alternatively, when both nutrients are regulated, there is an interaction between the regulatory mechanisms determining the intake of those nutrients and, thus, the array resembles a convex arc or a diagonal line (e.g.,  Lee et al., 2002; Raubenheimer & Simpson, 2003). On the other hand, if the intake of neither nutrient is regulated by a consumer, we could expect to observe a similar ingestion of food across diets irrespective of their nutrient content (Raubenheimer & Simpson, 1993; Simpson & Raubenheimer, 1995). Also, consumer’s decisions when facing a range of imbalanced foods can reveal which (or if) nutrients are prioritized during feeding, while the performance consequences of consuming those foods indicate the costs of such decisions (Simpson & Raubenheimer, 2011).

Marine mesograzers occur in high abundance associated with plant and algal hosts and may have impacts on benthic primary producers (Duffy & Hay, 2000; Poore et al., 2012; Whalen, Duffy & Grace, 2013). These small herbivores use macrophytes as both food and refuge (Duffy & Hay, 1991; Duffy & Hay, 1994; Poore & Steinberg, 1999; Sotka, 2007) and, since food value is an important factor driving the association of mesograzers with hosts (Duffy & Hay, 1991; Poore & Steinberg, 1999), exploring the nutrition of these consumers is relevant to understand their distribution. Also, there are some constraints to the feeding of these herbivores in the field, such as the temporal variation of the host (Duffy & Hay, 1991), the vulnerability of these consumers to predators and wave action (Duffy & Hay, 1994; Sotka, 2007; Lasley-Rasher et al., 2011) and interspecific competition (Beermann et al., 2018). Thus, exploring how mesograzers regulate the intake of nutrients, especially in cases of lacking nutritionally balanced foods, may contribute to understanding how these consumers interact with their environment.

Despite its analytical power, to our knowledge the geometric framework has not been applied to any marine mesograzer. Current understanding of the role of nutrients on marine mesograzers comes largely from univariate correlations of a particular nutritional constituent (e.g., nitrogen, carbon, or their ratio) with feeding behavior or its performance consequences (Nicotri, 1980; Duffy & Hay, 1991; Cruz-Rivera & Hay, 2001; Jormalainen, Honkanen & Heikkilä, 2001; McDonald & Bingham, 2010). When nutrients are evaluated individually (e.g., nitrogen content vs. feeding rate), the effect of nutritional multivariate complexity on feeding decisions by consumers is ignored (Simpson & Raubenheimer, 2011). Moreover, many studies also use the absolute amount of food consumed, rather than the ingestion of nutrients, as a proxy of the effect of nutrients on mesograzers (e.g.,  Cruz-Rivera & Hay, 2001; Sotka & Hay, 2002), which represents an indirect approach to understand how consumers actively regulate and select a balance of intake of nutrients.

Here, we investigated the nutrition of the herbivorous amphipod *Ampithoe valida* using algal foods varying in nutritional content and integrating feeding behavior and performance responses with nutrient intake. Specifically, we asked: (1) Does *A. valida* regulate the intake of nutrients when constrained to single foods varying in nutritional content (carbon, nitrogen, protein, and non-protein compounds) or when offered a cafeteria-style choice of these same foods? (2) What are the performance consequences of feeding diets varying in nutritional content?

## Material and Methods

### Organisms collection and maintenance

*Ampithoe valida* is a generalist mesograzer inhabiting seagrass and algal beds throughout the Atlantic and Pacific coasts of the United States (Duffy & Hay, 1994; Reynolds, Carr & Boyer, 2012) and Japan (E E Sotka, pers. obs., 2018), where it feeds readily on algae and seagrasses (Cruz-Rivera & Hay, 2000a; Douglass, Duffy & Canuel, 2011; Reynolds, Carr & Boyer, 2012). *Ampithoe valida* was obtained from *Ulva* spp. and other available algal hosts found in marinas and mudflats within Charleston Harbor, South Carolina (SC) (32.75°N, 79.90°W) (Field Permit #4447, Department of Natural Resources, Division of Marine Resources, Charleston, SC, USA). In the laboratory, amphipods were removed from algae and maintained individually in plastic cups with seawater (100 mL), under 12 h photoperiods and at a temperature of 22 °C. Amphipods were fed mainly *Ulva* spp. and kept in the laboratory for at least one week before being used in any assay. All assays presented below took place at Grice Marine Laboratory (College of Charleston, Charleston, SC, USA) during spring and summer 2016.

### Algal diets

For all assays, we used freeze-dried algal foods. The foods offered to *A. valida* in following assays represent a subset of a major collection of seaweeds used in a previous feeding study (see Demko et al., 2017) and, thus, with prior information about their nutritional content. The effect of toughness and morphology was eliminated by using freeze-dried instead of fresh diets, while metabolites are usually preserved in the freeze-dried state of seaweeds (e.g.,  Cronin & Hay, 1996; Taylor et al., 2003). Seaweeds were obtained from Charleston Harbor, Fiji (18.00°S, 179.00°E) and San Diego, California (32.72°N, 117.16°W) (Florida Special Activity License 06SR-971). We collected *Ulva* spp. at Charleston Harbor, (2014), Fijian seaweeds from shallow subtidal reefs (May to June 2014) and Californian seaweeds from a rocky intertidal shore (2013 and 2014; see Demko et al., 2017 for details). After collection, seaweeds were kept frozen in the laboratory and lyophilized.

All seaweeds were analyzed for ash-free dry mass (AFDM), carbon, nitrogen and protein content (% in dry mass; see Demko et al., 2017 for further details). Briefly, ash-free dry mass of freeze-dried tissue (AFDM) was determined from dried samples of seaweeds after placing them in a muffle furnace at 500 °C for 6–7 h. Protein content was measured using the methods of Bradford (1976). To estimate carbon and nitrogen content, freeze-dried seaweed samples were pulverized and analyzed using a NCS 2500 Series Elemental Analyzer (CE Instruments, Wigan, UK). We assumed AFDM content as all organic content, and non-protein content (including carbohydrates and lipids) was estimated as the organic content remaining after subtracting the protein content (per unit dry mass). The following algal species were chosen based on their differences in carbon to nitrogen ratio (from 10.4 to 108.1) and non-protein to protein ratio (from 8.3 to 66.1) (Table 1): *Egregia* sp., *Endarachne binghamiae*, *Gelidium coulteri*, *Hormophysa* sp., *Padina* sp., *Sargassum* sp., *Turbinaria* sp. and *Ulva* spp. Also, most of those seaweeds were chosen for being palatable to marine herbivores (Cruz-Rivera & Hay, 2001; Sotka, 2007; Rasher, Hoey & Hay, 2013; Demko et al., 2017). Although it would have been interesting using seaweeds without potent chemical defenses in the present study, this is not feasible when dealing with natural foods. Thus, it is likely that some if not all tested seaweeds have secondary metabolites, such as phenolic compounds, as reported by Demko et al. (2017). Because we used freeze-dried seaweed tissues, we cannot separate the effects of secondary metabolites (that might either increase or decrease feeding rates and performance) from nutritional content. Our interpretation reflects this limitation (see ‘Discussion’).

**Table 1:** Nutrient content of seaweeds (% per gram of freeze-dried tissue). AFDM, Ash-free dry mass. Data obtained from online resource provided by Demko et al. (2017) as a metadata (see DOI: http://doi.org/10.6084/m9.figshare.5514466.v1).

Seaweed	Site of collection	AFDM (%)	Carbon (%)	Nitrogen (%)	C:N	Non-protein (%)	Protein (%)	NP:P
*Egregia* sp.	San Diego	69.10	28.88	0.73	39.84	67.56	1.54	43.99
*Endarachne binghamiae*	San Diego	70.36	27.94	0.27	103.61	66.23	4.14	16.01
*Gelidium coulteri*	San Diego	83.81	33.77	1.52	22.30	82.56	1.25	66.07
*Hormophysa* sp.	Fiji	55.77	27.57	0.60	46.24	52.18	3.59	14.52
*Padina* sp.	Fiji	45.11	21.51	0.98	21.95	40.24	4.87	8.27
*Sargassum* sp.	Fiji	55.99	28.43	0.26	108.06	50.91	5.08	10.02
*Turbinaria* sp.	Fiji	64.70	28.33	0.58	48.87	61.12	3.57	17.12
*Ulva* spp.	Charleston	71.11	30.76	2.95	10.43	66.12	4.99	13.25

Typical tests of the geometric framework use artificial foods that vary in their ratio of two or more nutrients (e.g., Lee et al., 2002; Raubenheimer & Simpson, 2003; Hawlena & Schmitz, 2010). Such artificial foods are often prepared by adding individual nutrients, such as protein (a mix of casein, peptone and albumen), carbohydrate (a mix of sucrose and white dextrin), linoleic acid, cholesterol, vitamins, and salts, into an agar matrix. However, as argued previously (Forbey et al., 2013), leaching generates poor residence time of nutrients within the aqueous environment. A similar issue has been reported by Carefoot (1980), when investigating the nutrition of *Aplysia*. One of the main concerns is the possibility that different nutrients have different degrees of diffusion in water (Carefoot, 1980). Ideally, it would be useful to seal such nutrients into the artificial foods. On the other hand, as pointed out by Carefoot (1980), the issue of leaching of nutrients can be minimized when diets are offered as whole meals (i.e., fish meal, soybean meal), probably because all nutrients are bound in a single matrix. In fact, many studies on nutritional aspects of aquatic invertebrates have used complete foods (e.g., algae, animal matter), or a combination of those, varying in nutrient content in order to investigate the importance of nutrients on the behavior and performance of consumers (Cruz-Rivera & Hay, 2000b; Cruz-Rivera & Hay, 2003; Heflin et al., 2016). While leaching of nutrients may not be totally prevented when using freeze-dried algal material, it is likely that the integrity of most cell walls and membranes minimizes its effect, especially when compared to the dissolution of solubilized nutrients in agar-based foods. The complexity of nutritional composition represented by these eight seaweeds thus serves as an initial test of the relative importance of nutritional concerns in driving feeding behavior and juvenile fitness.

### Feeding experiments: preparation of algal diets and general methods

To evaluate the ingestion of food and nutrients by *A. valida* in a variety of diets, we carried out no-choice and choice feeding assays with freeze-dried algal foods. The use of natural foods in such state to investigate the feeding of marine herbivores has been successfully applied before (Hay, Kappel & Fenical, 1994; Cruz-Rivera & Hay, 2000a). Usually, ground powdered food mixed with molten agar is poured onto a screen mesh and then the number of squares cleared over time is considered as a measurement of consumption (Hay, Kappel & Fenical, 1994). Since herein we were interested in estimating the quantity of nutrients ingested, rather than solely testing which food is more consumed, we adapted this common methodology to be able to measure the food mass change throughout the experiment and, consequently, estimate the nutrient intake. Also, since nutrient composition can vary among algal individuals (Cronin & Hay, 1996); (Taylor, Sotka & Hay, 2002; Taylor et al., 2003), using a same source of freeze-dried algal foods for all experiments is likely to maintain a similar nutritional composition across the feeding assays and, thus, avoids effects related to the natural variation of nutrients in the food.

For feeding assays, we ground the freeze-dried seaweeds to a fine powder using a Wiley Mill. Ground powdered seaweed (2 g) was mixed with 7 mL distilled water and then poured into molten agar (0.36 g to 18 mL distilled water). The resulting mixture was added to pre-weighed Eppendorf lids to solidify. All lids with food were weighed (wet mass) before being offered to amphipods. Also, a same number of cups with algal food, but no amphipods, were used to control mass changes in food not related to herbivory. After the experiment, lids were removed from cups, carefully washed with distilled water, dried at 60 °C until reaching a constant mass and then reweighed. To estimate the initial dry mass of foods used in the assays, the same number of lids with food were prepared initially and the wet mass was measured. Afterwards, these foods were dried at 60 °C to a constant mass and then reweighed. Using equations obtained from linear regressions between wet and dry mass for each algal food, we could estimate the initial dry mass of foods in herbivory and control cups. Finally, the consumption (mg) in cups with herbivores was expressed as the difference between initial and final dry mass after correcting it by mass change in control cups (see Cronin & Hay, 1996). Before analysis, negative values of corrected mass change (39 out of 211 lids for no-choice experiment and 29 out of 102 lids for choice experiment) were assumed as zero (i.e., no consumption). Furthermore, for each nutrient, we estimated the ingestion (mg in dry mass) by considering the content (%) present in each algal food and the total amount of food ingested (excluding the agar portion).

### No-choice feeding experiments

To test how *A. valida* behaves when constrained to single foods varying in nutritional content, we carried out a no-choice feeding experiment with adults using seven algal foods: *Egregia* sp., *Endarachne binghamiae*, *Hormophysa* sp., *Padina* sp., *Sargassum* sp., *Turbinaria* sp. and *Ulva* spp. We split the experiment in three trials, each one with 10 replicates of each diet for herbivory and control cups. Individual amphipods were kept in seawater (∼100 mL) to which one food type was added. All amphipods were removed after approximately 85 h. Amphipods were then frozen and preserved in ethanol, and amphipod size (considered as the length from the anterior region of the head to the distal region of uropod 3) was measured using ImageJ software after taking digital photos in a microscope. The size of amphipods used in the no-choice experiment did not differ between the three independent trials (ANOVA, *F*(2, 164) = 2.49, *P* = 0.086) nor diets (ANOVA, *F*(6, 164) = 0.77, *P* = 0.595). Replicates with dead amphipods at the end of trials were excluded from analysis.

Comparison of the mass change between foods with and without amphipods using unpaired t-tests indicated significant grazing of all diets in at least two of three trials, with the exception of *Turbinaria* (Table S1). *Ampithoe valida* consumed *Turbinaria* in only one of the three trials and this consumption was minimal; thus, we did not further consider this seaweed in either the multiple-choice assays (see below) nor during subsequent analysis. To compare the consumption of algal foods by *A. valida* among diets, we used mixed linear model treating diet as a fixed factor and trial as a random factor. We tested the contribution of the factor ‘diet’ for the model by comparing the full model with the reduced one (without ‘diet’) using Likelihood Ratio Test in the *lme4* package of R (Bates et al., 2015).

### Multiple-choice feeding experiments

To investigate the feeding preference of *A. valida* and to assess nutrient intake when offered a choice of foods, we carried out a multiple-choice feeding experiment using six algal foods: *Egregia* sp., *Endarachne binghamiae*, *Hormophysa* sp., *Padina* sp., *Sargassum* sp. and *Ulva* spp. We split the experiment in two trials, each one with 10 replicates for herbivory and control cups. Amphipods were kept individualized in cups with seawater (∼200 mL) and six foods for 86 h. Amphipods used in the choice experiment did not differ between trials (ANOVA, *F*(1, 15) = 3.21, *P* = 0.094). To assess whether amphipods made feeding choices, we performed the Hotelling *T*^2^ test, followed by pairwise comparisons on the proportional consumption of each food relative to the total mass consumed (see Lockwood, 1998).

### Analysis of behavioral regulation

We inferred behavioral regulation of a particular nutrient using three tests. First, we assessed whether rates of nutrient intake are similar across foods that vary in nutrient content (Raubenheimer & Simpson, 1993; Raubenheimer et al., 2005; Harrison et al., 2014). Second, we assessed whether rates of nutrient intake on a single food are similar to intake rates when offered multiple foods that vary in nutrient content (Raubenheimer & Simpson, 1993; Trumper & Simpson, 1993). For that, we compared the intake rate of each nutrient type on each single diet with the summed intake rates of that nutrient type in multiple choice assays. Because we used smaller amphipod individuals in choice assays relative to amphipods in no-choice assays (ANOVA, *F*(1, 172) = 15.59, *P* = 0.0001), we standardized feeding rates by amphipod size (mg mm^−1^). We then used one-way parametric ANOVA to compare the intake rates of carbon and non-protein components across the six single diets and the summed rate in choice assays. For nitrogen and protein intake, we could not meet the ANOVA’s assumptions, and thus we used generalized linear model (GLM) with quasi-Poisson distribution to analyze the data. Third, we tested the prediction that when consumers are behaviorally regulating nutrient intake, variability in nutrient content among food types will be greater than the variability for nutrient intake values. To do this, we compared the coefficients of variation, or CV (i.e., standard deviation divided by the overall mean), which creates estimates of variability that are independent of measurement type (Sokal & Rohlf, 1995). We calculated CV of the four nutrient types among algal foods (Table 1) and among mean intake values during no-choice assays. Comparisons of such variability between content and intake values were performed using an *F*-test with log-transformed data (*X* + 1) (Lewontin, 1966; Sokal & Rohlf, 1995). To test for the possibility that our estimates of CVs for nutrient intake were biased when we imputed zeros for negative values (see above), we repeated this test using CVs calculated with negative values.

### Performance experiments

To test the performance consequences for *A. valida* when constrained to single foods varying from each other in nutritional content, we carried out a performance experiment with juveniles under one of the following diets (*n* = 29 per diet): *Egregia* sp., *Endarachne binghamiae*, *Gelidium coulteri*, *Hormophysa* sp., *Padina* sp., *Sargassum* sp., *Turbinaria* sp., *Ulva* spp., agar and no food (control). Juveniles were obtained from field-collected ovigerous females. Females were kept individualized in cups with seawater until juveniles hatched. From each female, we used 1 to 2 juveniles per treatment. Juveniles were kept individualized in cups with seawater (∼200 mL) and checked every day for dead individuals, which were preserved in ethanol for further analysis. Also, throughout the experiment, some females ovulated and, thus, presented unfertilized eggs (hereafter, referred as eggs) in a brood pouch. Because juveniles were separated, such eggs do not represent zygotes, but they have been used as an estimative of reproductive potential in several studies (Duffy & Hay, 1991; Cruz-Rivera & Hay, 2001; Sotka & Hay, 2002; Taylor & Brown, 2006). When a female presented eggs, it was preserved in ethanol for further analysis. Foods were prepared and offered as in the feeding assays described above. Every 4 to 5 days, food was changed, cups were cleaned, and water was replaced. Although care was taken, eventually a few individuals were missed throughout the experiment and, thus, the initial number of replicates varied from 27 to 29 individuals per treatment. After 40 days, surviving individuals (i.e., males and females without eggs) were frozen and then preserved in ethanol. Individuals were measured as described above. Survivorship (%) was considered as the proportion of individuals alive at each day until the first female presented eggs. Because we deliberately sacrificed females with eggs to count the number of eggs, we cannot consider this death as an effect of the treatment and, thus, in order to standardize across the treatments, all deaths after the first female presented eggs were not considered for the survivorship analysis. For growth (mm day^−1^), we considered only individuals that survived after all amphipods in the control (no food) had perished. As reproduction variables, we counted the number of eggs per female and the time to ovulation (days).

To compare survivorship among diets, we used the *survival* package in R (Therneau & Grambsch, 2000) to perform a log-rank test with all treatments, followed by pairwise comparisons to assess *post hoc* differences (Fox, 2001). To test growth among diets, we performed GLM with quasi-Poisson distribution. To compare the number of eggs per female and the time to ovulation among diets, we used one-way ANOVA. Also, to explore possible trade-offs between the performance traits, we generated correlations among mean values of growth, number of eggs and the survivorship at the day the first female presented eggs. For ANOVAs, the assumptions of normality and homogeneity of variances were verified using graphs. Tukey’s test was used to explore differences between groups after the effect of a factor has been detected. For all ANOVAs, GLMs, correlation analyses, tests of variance and Tukey’s tests, we used functions in *multcomp* (Hothorn, Bretz & Westfall, 2008) and *stats* packages in R 3.2.3 (R Core Team, 2015).

## Results

### Feeding experiments

**Figure 1:**
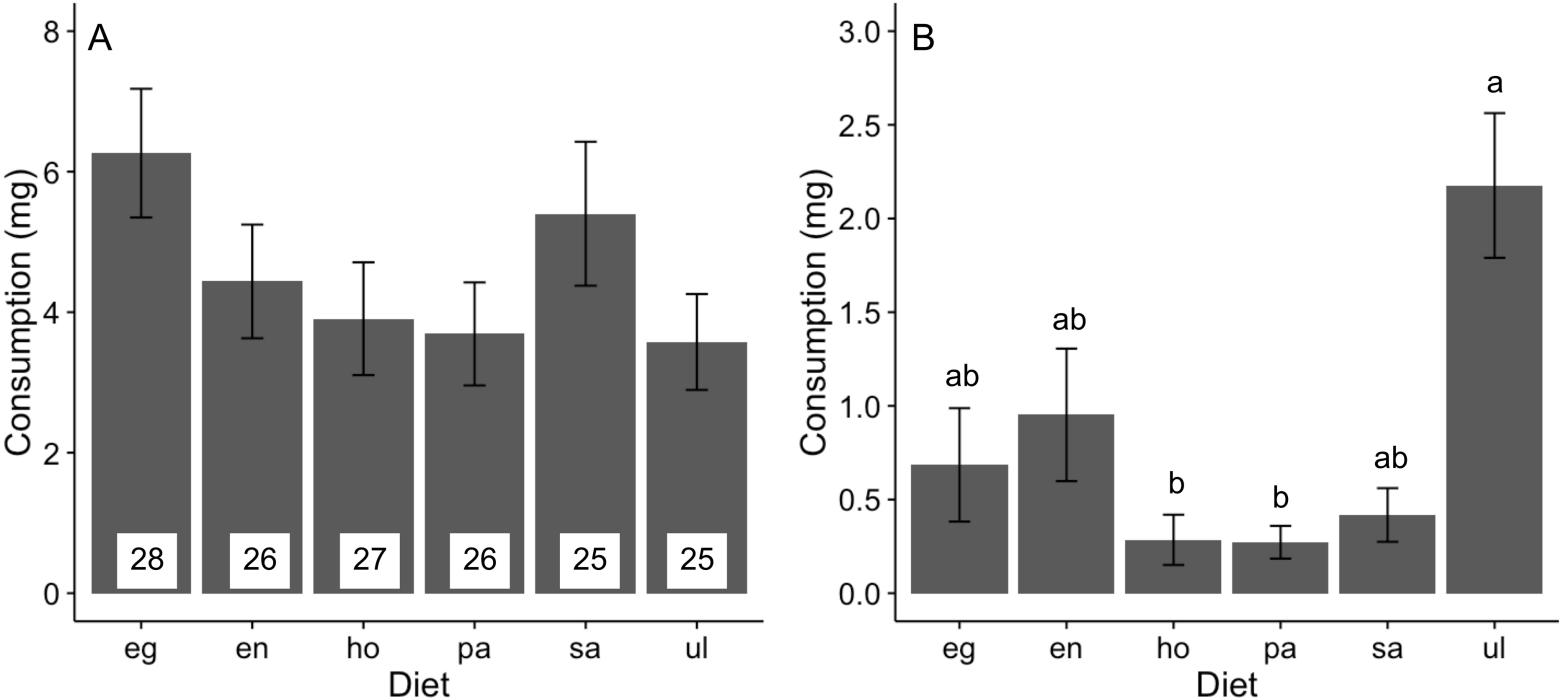
Consumption (mean ± SE) of algal diets by *A. valida*. (A) No-choice and (B) choice feeding experiments. For both experiments, data represents the summary across all trials. In (A), number of replicates per diet is indicated within bars. In (B), *n* = 17. Differences in letters between bars indicate significant difference (*P* < 0.05). eg, *Egregia* sp.; en, *Endarachne*; ho, *Hormophysa* sp.; pa, *Padina* sp.; sa, *Sargassum* sp.; ul, *Ulva* spp.

Feeding rates of *A. valida* did not differ among the six diets (likelihood ratio test, *χ*^2^ = 9.58, *P* = 0.088), although there was a trend for higher consumption of *Egregia* and *Sargassum* relative to other foods (Fig. 1A). The relative consumption rates of seaweeds were similar across trials (Fig. S1). In the choice feeding experiment, *A. valida* avoided *Hormophysa* and *Padina,* readily consumed *Ulva,* and had intermediate feeding rates on the remainder (Hotelling’s *T*^2^ test, *T*^2^ = 53.20, *F*(5, 12) = 7.98, *P* < 0.05; Fig. 1B). The pattern of consumption by *A. valida* was similar among trials (Fig. S2). We note that feeding rates by *A. valida* were statistically indistinguishable when isolated on a single diet or when offered multiple foods (ANOVA, *F*(6, 167) = 1.26, *P* = 0.277).

### Nutrient intake in feeding experiments

**Figure 2:**
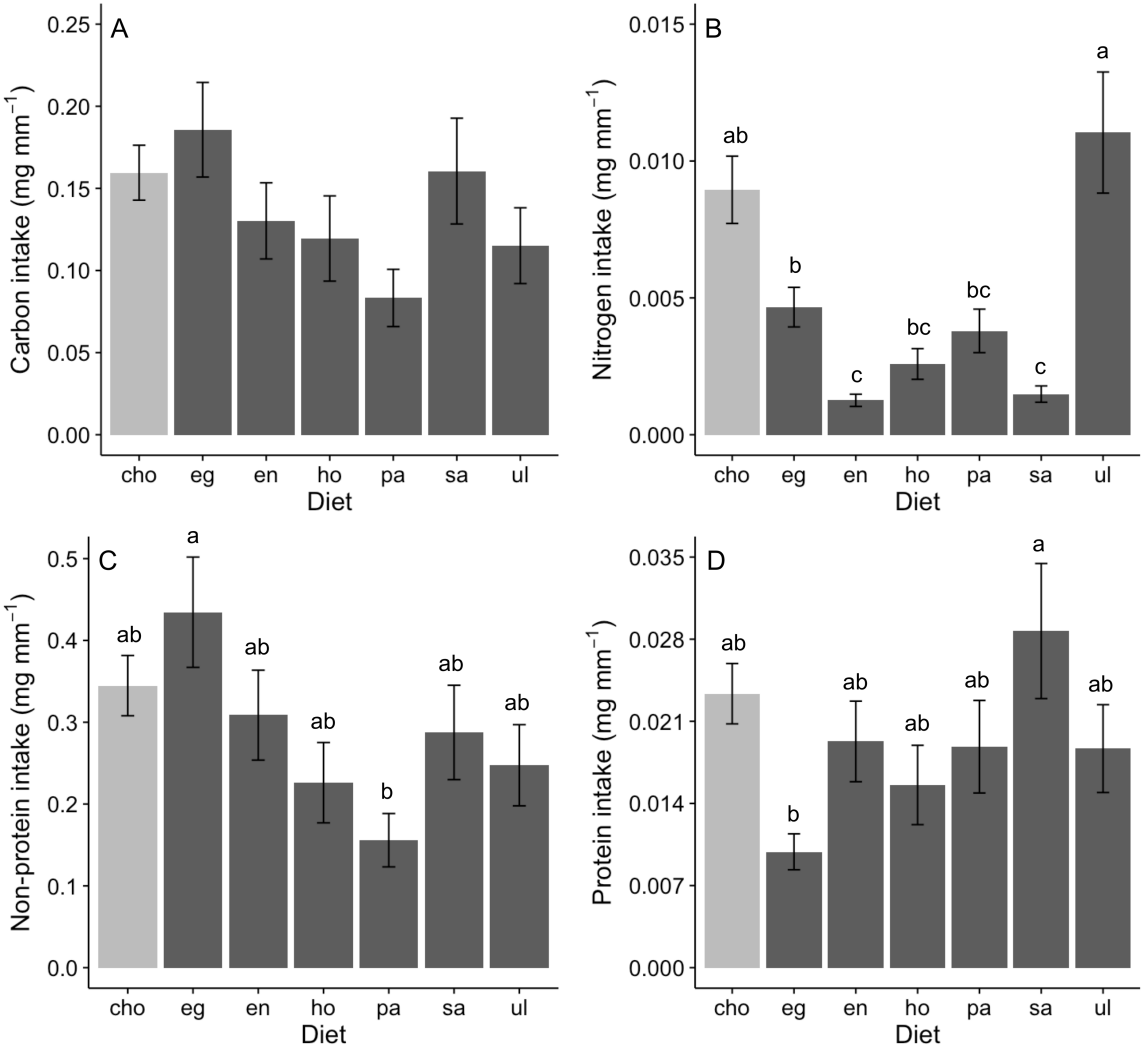
Nutrient intake (mean ± SE) (mg per amphipod length in mm) by *A. valida* in single and choice algal diets. (A) Carbon intake; (B) Nitrogen intake; (C) Non-protein intake; (D) Protein intake. Differences in letters between bars indicate significant difference (*P* < 0.05). “cho” represents the total nutrient consumption in the choice diet regardless the algal identity. See Fig. 1 for abbreviations and number of replicates.

Assessing behavioral regulation of nutrients requires analysis of the intake rate of particular nutrients (i.e., multiplying nutritional content of a food item by feeding rate; see predictions outlined in *Analysis of behavioral regulation*). Carbon intake rates did not differ across foods in no-choice assays, nor when rates in no-choice and choice assays were directly compared (ANOVA, *F* (6,167) = 1.93, *P* = 0.078; Fig. 2A). In contrast, intake rates of nitrogen (GLM; Deviance = 0.409, Residual deviance = 0.684, *P* < 0.0001; Fig. 2B), non-protein components (ANOVA, *F* (6,167) = 2.03, *P* = 0.008; Fig. 2C) and protein (GLM; Deviance = 0.289, Residual deviance = 3.078, *P* = 0.012; Fig. 2D) significantly differed among diets. Nitrogen intake was greatest when offered *Ulva* or a mix of diets (“cho”) and was lowest on *Endarachne* and *Sargassum* (Fig. 2B). Non-protein intake was greatest when offered *Egregia*, was lowest when offered *Padina* diet, and was statistically indistinguishable among remaining diets, including the mix of diets (Fig. 2C). Protein intake was highest on *Sargassum*, lowest on *Egregia* and statistically indistinguishable among remaining diets, including the mix of diets (Fig. 2D).

**Figure 3:**
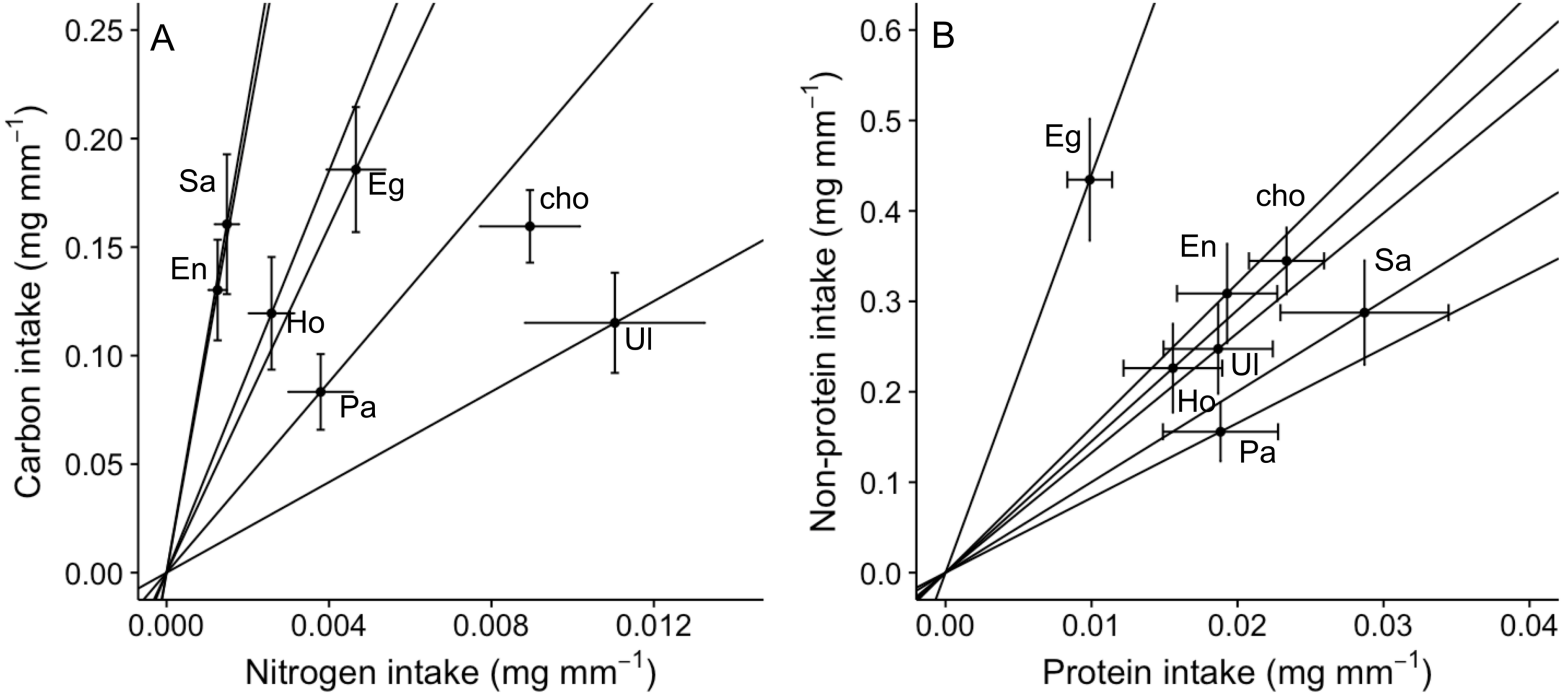
Bi-variate representation of nutrient intake (mean ± SE) (mg per amphipod length in mm) by *A. valida* across single and choice diets. (A) Carbon and nitrogen intake; (B) non-protein and protein intake. See Fig. 1 for abbreviations and number of replicates. In (A) and (B), “cho” represents the total nutrient consumption in the choice diet regardless the algal identity. Slopes represent trajectories in which consumers are constrained when fed single diets.

An analysis of the coefficient of variation (CV) in nutritional intake within no-choice assays was significantly lower than the CV in nutritional content for nitrogen (*P* < 0.001; Table 2A). In contrast, CV in nutritional intake was higher than CV in nutritional content for carbon (*P* = 0.012) and non-protein content (*P* = 0.041) (Table 2A). Also, a visual inspection of a bivariate plot between nitrogen and carbon intake rates (Fig. 3A) indicates that amphipods within the choice assay (‘cho’) have among the greatest carbon and nitrogen rates, combined. That is, while some single foods provided greater nitrogen intake (*Ulva*) or carbon intake (*Egregia*), the choice assay has among the greatest values for both carbon and nitrogen. Similarly, amphipods within the choice assay have among the greatest protein and non-protein intake, relative to the single food assays (Fig. 3B). We note that these CV results are consistent when we used the negative consumption values (see *Methods*) for carbon, nitrogen and non-protein components (Table 2B). However, for protein, the intake CV is greater than content CV when using imputed zeros, while intake CV is lower than content CV when using raw data (Table 2B). Because of the low signal and dependency of the results on how we treat the dataset, we interpret the behavioral regulation of protein as equivocal using this test.

**Table 2:** Measure of variability for values of content and intake of nutrients. (A) values of intake with zero adjustment; (B) values of intake without zero adjustment.

Nutrient	Coefficient of variation		*F*-test for equality of variances	
	Content	Intake	*F* (5,5)	*P*
**A**				
Carbon	11.43	27.27	13.99	0.012
Nitrogen	104.88	87.67	13,708	<0.001
Non-protein	19.48	33.93	7.84	0.041
Protein	33.53	33.13	3,094	<0.001
**B**				
Carbon	11.43	29.12	12.919	0.014
Nitrogen	104.88	89.79	13,578	<0.001
Non-protein	19.48	35.80	7.38	0.047
Protein	33.53	33.92	3,135	<0.001

### Consequences for juvenile performance

**Figure 4:**
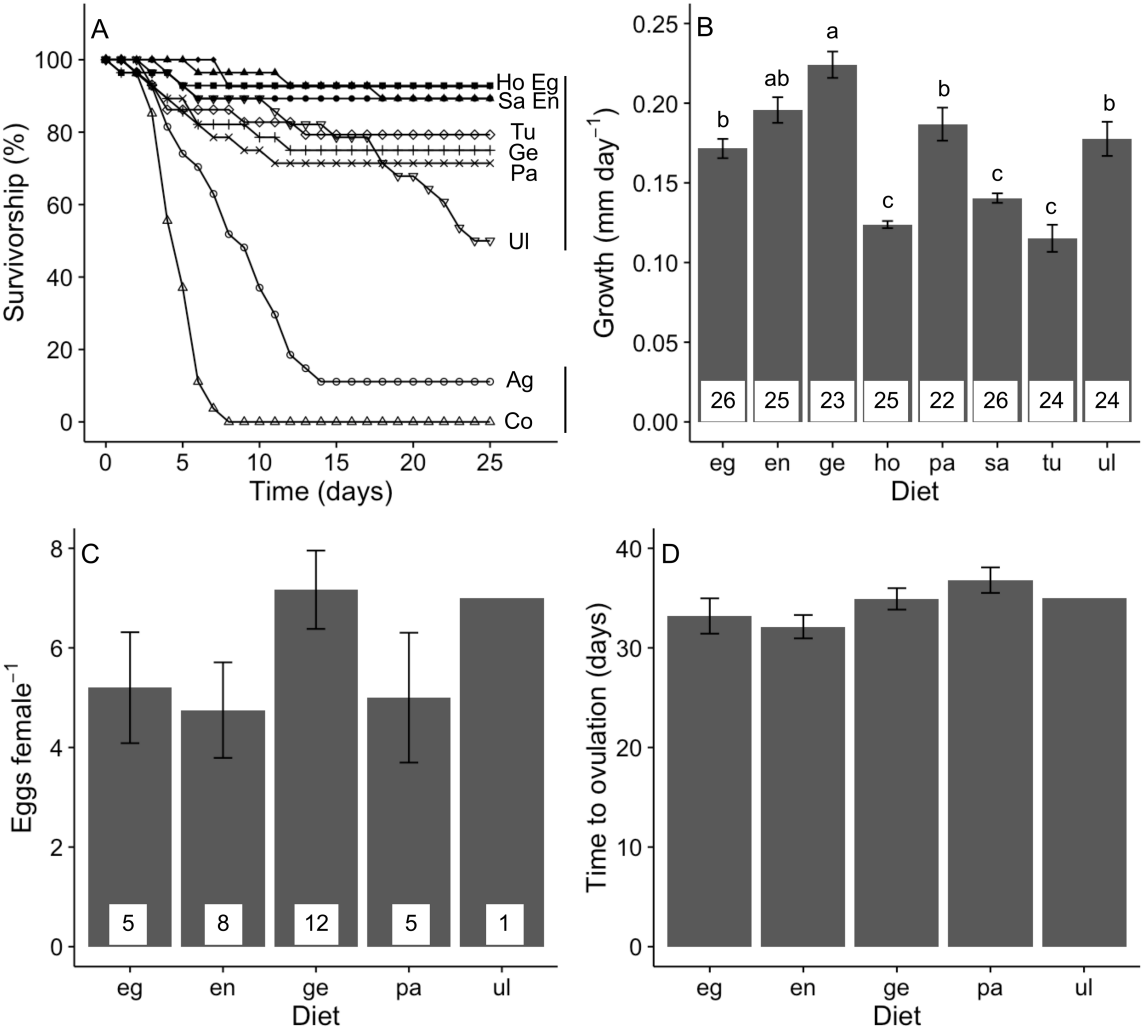
Performance of *A. valida* fed single algal diets. (A) Survivorship (%) (*n* = 27–29); (B) growth (mean ±  SE); (C) number of eggs per female (mean ± SE); (D) time to ovulation (mean ± SE). Co, control diet, Ag, agar diet (see Fig. 1 for abbreviations). In (A), diets sharing a same vertical solid line are not significant different. In (B), differences in letters between bars indicate significant difference (*P* < 0.05). Number of replicates per diet is indicated within bars.

Our treatments significantly affected the 25-day survival of *A. valida* juveniles (log-rank test, *χ*^2^ = 201, *P* < 0.05; Fig. 4A). This was explained by the lower survivorship when isolated onto agar and no-food control treatments. When only amphipods isolated on algal diets was considered, we detected no significant difference in survival, although there was a non-significant trend for juveniles fed *Ulva* to have lower survival.

Algal diet affected the growth of *A. valida* (GLM, Deviance = 1.446, Residual deviance = 1.370, *P* <  0.0001). Amphipods had a higher growth rate consuming *Gelidium*, *Endarachne*, an intermediate growth under *Egregia*, *Ulva* and *Padina* diets and a lower growth when fed *Sargassum*, *Hormophysa* and *Turbinaria* (Fig. 4B). *Ampithoe valida* females became reproductive only when isolated on *Gelidium*, *Endarachne*, *Egregia*, *Padina* and *Ulva* (Fig. 4C). On *Ulva* diet, only one female presented eggs. When the *Ulva* treatment is ignored, we found no significant difference among diets in the number of eggs per female (ANOVA, *F*(3, 26) = 1.63, *P* = 0.206; Fig. 4C) nor any significant difference in time to ovulation (ANOVA, *F*(3, 26) = 2.12, *P* = 0.123; Fig. 4D). There was no significant correlation between survivorship and growth ( *r* =  − 0.31, *n* = 8, *P* = 0.450) neither between survivorship and number of eggs (*r* =  − 0.55, *n* = 8, *P* = 0.157). However, there was a strong positive correlation between growth on a food type and number of eggs produced (*r* = 0.90, *n* = 8, *P* = 0.002).

## Discussion

Typical studies on the nutritional ecology of marine herbivores correlate adult feeding rates or juvenile fitness on a single diet with plant traits such as carbon, nitrogen, organic content and/or protein (Duffy & Hay, 1991; Cruz-Rivera & Hay, 2001; McDonald & Bingham, 2010), and do not directly compare nutrient content with nutrient intake (e.g., Table 2). These approaches are limited because consumers are ingesting multiple nutrients simultaneously, and there are constraints when trying to maximize the use of any one nutrient type. Here, we were inspired by the geometric framework to compare nutrient content and intake values using a series of feeding and juvenile performance assays. We discuss these results below.

### Behavioral regulation of nutrients

Behavioral regulation of a particular nutrient can be indicated by several criteria: (1) rates of nutrient intake are similar across foods that vary in nutrient content (Raubenheimer & Simpson, 1993; Raubenheimer et al., 2005; Harrison et al., 2014), (2) rates of nutrient intake on a single food is similar to intake rates when offered multiple foods that vary in nutrient content (Raubenheimer & Simpson, 1993; Trumper & Simpson, 1993), and (3) variation in nutrient content across foods (as measured by coefficient of variation, or CV) is greater than the CV for nutrient intake when those foods are offered singly to a consumer. For the generalist consumer *Ampithoe valida*, none of the four nutrient types (carbon, nitrogen, protein, non-protein) confirmed all three predictions for behavioral regulation, suggesting either that our methods are insufficient to detect behavioral regulation, weak regulation, or both.

We found little to no empirical support in the generalist consumer *Ampithoe valida* for regulation of protein and non-protein components (criterion 2). Non-protein content was considered as all organic content remaining after subtracting the protein content (per unit dry mass), and it includes carbohydrates and lipids. The literature on geometric framework of nutrition largely focuses on the bivariate relationships of proteins and carbohydrates, as these plots consistently show actively regulation by terrestrial and aquatic consumers (Lee et al., 2002; Raubenheimer et al., 2005; Heflin et al., 2016). Proteins are mainly related to growth and reproduction of organisms, while carbohydrates are used as energy for metabolic activities (Joern & Behmer, 1997; Raubenheimer & Simpson, 2003), although some interchangeability in function can be observed among both macronutrients (e.g., protein being used as energy; Raubenheimer & Simpson, 2003). Also, protein content has been positively correlated with feeding behavior and/or performance of mesograzers (Duffy & Hay, 1991; Cruz-Rivera & Hay, 2000a; Cruz-Rivera & Hay, 2001; Duarte et al., 2011).

We found greater empirical support for behavioral regulation of carbon (criteria 1 and 2) and nitrogen (criterion 3) intake. Nitrogen is widely thought to be a limiting nutrient for herbivores, not only because it usually occurs in low concentration in plants relative to the amount required by these consumers, but also due to its great inter- and intra-plant variation (Mattson, 1980). While nitrogen content is often used as a proxy for food protein levels (Renaud, Thinh & Parry, 1999; Renaud & Luong-Van, 2006), here, nitrogen content was poorly related to protein content (e.g., *Sargassum* has high levels of protein, but low nitrogen content), as in other studies (Kaehler & Kennish, 1996; Cruz-Rivera & Hay, 2001). This may reflect the use of nitrogen in other non-protein components present in seaweeds (Lourenço et al., 2002). Also, although the carbon intake by *A. valida* satisfied both criteria 1 and 2, there was an increase in variation from content to intake of such element (the opposite result of criteria 3 expectations). So, the conclusion of some behavioral regulation of carbon by *A. valida*, should be taken with caution. Also, carbon can constitute nutritional components, such as starch, as well as non-nutritional compounds, such as cellulose, which can blur the interpretation about behavioral responses of consumers to that element (Raubenheimer, Simpson & Mayntz, 2009). If we assume that the CV approach is the most powerful test of regulation (Table 2), then regulation of nitrogen intake is stronger than behavioral regulation of carbon, protein and non-protein by *A. valida*.

### Post-ingestive consequences

In theory, there will be a fitness consequence when prioritizing protein intake over non-protein intake, as non-protein compounds such as carbohydrates are crucial in building and maintaining organisms. Surprisingly, *A. valida* seems to avoid such fitness costs. An under-ingestion of non-protein was evident for individuals fed *Padina* (intake nearly one-half of that in choice diet), while an over-ingestion was observed in *Egregia* diet (intake about 25% higher than that in choice diet; Fig. 3B). However, the survival of *A. valida* juveniles was similar among all diets. Also, growth and reproduction of *A. valida* fed *Egregia* and *Padina* were comparable to that in other foods.

These results suggest that post-ingestive mechanisms in *A. valida* can overcome the excess and deficit of nutrients, as has been observed for other consumers (Zanotto, Simpson & Raubenheimer, 1993; Lee et al., 2002; Raubenheimer & Simpson, 2003). The insect *Locusta migratoria* can overcome the excess of macronutrients post-ingestively, as similar growth is reached by individuals raised on diets varying in protein and carbohydrate content (Zanotto, Simpson & Raubenheimer, 1993). In turn, the insect *Schistocerca gregaria* can use protein as energy metabolism to deal with the deficit of carbohydrate acquired from diet (Raubenheimer & Simpson, 2003). Likewise, the freshwater amphipod *Gammarus pulex* can decrease its respiration rate when fed poor-quality foods, reducing energy expenditure and, thus, compensating for lower energy intake (Graça, Maltby & Calow, 1993). Although performance variables represent the ultimate consequences of behavioral and physiological mechanisms and, thus, they can bring important insights about the processes involved in the regulation of nutrient intake, further studies measuring the efficiency of utilization of ingested nutrients (i.e., relationship between amount consumed and amount retained or converted to growth) (e.g., Lee et al., 2002; Raubenheimer & Simpson, 2003) are necessary to a better understanding on post-ingestive regulation in *A. valida*.

*Ampithoe valida* juveniles had similar survival rates across all algal diets. However, when other performance variables are considered, such as growth and reproduction, *A.  valida* performed better on some foods (e.g., *Endarachne*, *Egregia* and *Padina*) than on others (e.g., *Sargassum* and *Hormophysa*), despite a similar intake of macronutrients. In fact, there was no relationship between survivorship and growth neither between survivorship and number of eggs, while growth and number of eggs were positively correlated. The disagreement among performance variables reinforces the importance of measuring more than one variable when investigating the nutrition of consumers, as argued previously (Simpson & Raubenheimer, 1995).

### A role for chemical defense and other plant traits

Although *A. valida* has shown strong preference towards *Ulva*, the long-term effect of consuming that food did not result in a better performance for this mesograzer than other less preferred foods (e.g., *Padina* and *Egregia*), suggesting that feeding preference and performance of marine herbivores are not positively related. In some marine herbivores, preference and performance are positively related (e.g., Vadas, 1977; Poore & Steinberg, 1999; Taylor & Brown, 2006; Aquilino, Coulbourne & Stachowicz, 2012). However, this is not always the case for marine mesograzers. The herbivorous isopod *Idotea baltica* shows preference for phlorotannin-rich seaweeds, such as *Fucus vesiculosus*, but this causes a reduced fitness when raised in such algal hosts (Jormalainen, Honkanen & Heikkilä, 2001). Also, Cruz-Rivera & Hay (2001) reported the generalist herbivorous *Ampithoe longimana* did not have a better performance (survival, growth and reproduction) on preferred foods (e.g., *Dictyota menstrualis*) than avoided foods (e.g., *Sargassum filipendula*).

Several mechanisms can yield a mismatch between feeding preference and performance. The first is compensatory feeding, which enables consumers to achieve similar performance outcomes among diets with different nutritional values (Cruz-Rivera & Hay, 2001). Second is the presence of seaweed metabolites that have only post-ingestive effects. One example may be *Ulva,* which generates DMSO (dimethylsulfoniopropionate) that is rapidly converted to metabolites that can act as defenses against herbivory (Van Alstyne et al., 2001; Van Alstyne, Pelletreau & Kirby, 2009). Therefore, although there appears to be a feeding preference of *A. valida* towards *Ulva*, the long-term effect of consuming such food may impose fitness costs related to the presence of chemical defenses. Also, it could be argued that the strong preference of *A. valida* towards *Ulva* may occur because such herbivore is familiar with this food at its habitat in Charleston Harbor, while the remaining foods represent a novelty, since they were collected in other places. However, it is important to note that (1) *A. valida* is a generalist herbivore, being able to consume a variety of algal foods (Cruz-Rivera & Hay, 2000a; Douglass, Duffy & Canuel, 2011; Reynolds, Carr & Boyer, 2012); (2) although there was a possible effect of novelty in the choice feeding experiment, it does not seem to be the case in the no-choice feeding experiment (in which amphipods consume *Ulva* and other seaweeds at similar rate) and in the performance experiment (in which juveniles performed similarly or better in other seaweeds than in *Ulva*).

Furthermore, *A. valida* avoided *Turbinaria* during no-choice experiments, although that food has similar nutritional composition as other readily consumed algae (e.g., *Endarachne*, *Hormophysa*). It suggests that the consumer may be also responding to other nutrients (e.g., vitamins or mineral salts; (Dadd, 1961; House, 1961; Trumper & Simpson, 1993) and/or other food properties not investigated here.

## Conclusions

The nutrition of marine mesograzers has been investigated by correlating the amount of food ingested and performance consequences with the food nutritional content (Duffy & Hay, 1991; Cruz-Rivera & Hay, 2001; McDonald & Bingham, 2010). Such an approach is an indirect way of investigating the role of nutrients on mesograzers, as it does not consider that consumers can actively regulate and select a balance of ingestion of two or more nutrients simultaneously. Herein, using criteria from the geometric framework and comparing the variability among nutrient content and intake values, we had some evidence that a generalist mesograzer more strongly behaviorally regulates and maximizes ingestion of carbon and nitrogen rather than protein and non-protein components. Over the developmental lifespan of *A. valida,* these preferences did not strongly predict survivorship, growth nor reproductive output. We find that an integrative approach considering the intake of multiple nutrients can bring valuable insights regarding the mechanisms underlying the feeding behavior of mesograzers and its performance consequences and, thus, should be considered in further studies on the nutrition of marine consumers.

##  Supplemental Information

10.7717/peerj.5929/supp-1 Figure S1 Consumption (mean ±SE) of algal diets by *Ampithoe valida* in no-choice experiment (A) Data represents a summary across all trials; (B) First trial; (C) Second trial; (D) Third trial. eg, *Egregia* sp.; en, *Endarachne*; ho, *Hormophysa* sp.; pa, *Padina* sp.; sa, *Sargassum* sp.; tu, *Turbinaria*; ul, *Ulva* spp.

10.7717/peerj.5929/supp-2 Figure S2 Consumption (mean ±SE) of algal diets by *Ampithoe valida* in choice experiment (A) Data represents a summary across all trials; (B) First trial; (C) Second trial. eg, *Egregia* sp.; en, *Endarachne*; ho, *Hormophysa* sp.; pa, *Padina* sp.; sa, *Sargassum* sp.; ul, *Ulva* spp.

10.7717/peerj.5929/supp-3 Table S1 *t* test for unequal variances comparing food mass change between grazed and ungrazed cups by trial in no-choice feeding experiment

10.7717/peerj.5929/supp-4 Supplemental Information 1 Raw data for feeding assays

10.7717/peerj.5929/supp-5 Supplemental Information 2 Raw data for performance experiment

## Additional Information and Declarations

### Field Study Permissions

The following information was supplied relating to field study approvals (i.e., approving body and any reference numbers): Field experiments were approved by Field Permit #4447, Department of Natural Resources, Division of Marine Resources, Charleston, SC. For the tropical seaweeds, organisms were collected with Florida Special Activity License 06SR-971 to Valerie Paul (Smithsonian Institution).

### Data Availability

The following information was supplied regarding data availability: The raw data are provided in the Supplemental Files.
