# Supplementary material for: Nutrition of marine mesograzers: integrating feeding behavior, nutrient intake and performance of an herbivorous amphipod
Source: PeerJ. 2018 Nov 9;6:e5929. doi: 10.7717/peerj.5929 (PMC6231427; doi:10.7717/peerj.5929)
Supplement: Figure S2 — (A) Data represents a summary across all trials; (B) First trial; (C) Second trial. eg, Egregia sp.; en, Endarachne; ho, Hormophysa sp.; pa, Padina sp.; sa, Sargassum sp.; ul, Ulva spp. [file peerj-06-5929-s002.pdf]

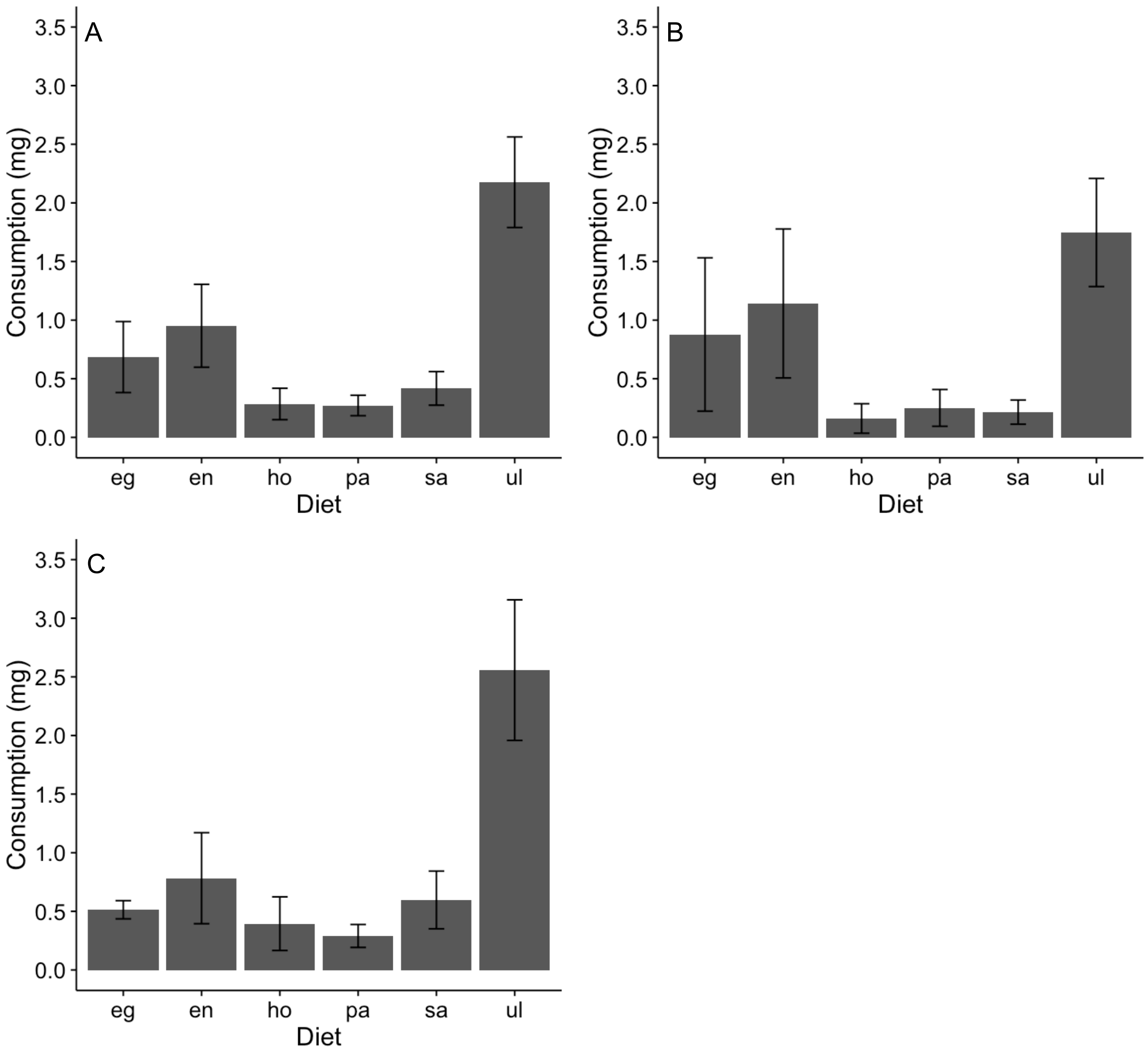

Figure S2. Consumption (mean±SE) of algal diets by Ampithoe valida in choice experiment. (A) Data represents a summary across all trials; (B) First trial; (C) Second trial. eg = Egregia sp., en = Endarachne, ho = Hormophysa sp., pa = Padina sp., sa = Sargassum sp., ul = Ulva spp.
