# Supplementary material for: Nutrition of marine mesograzers: integrating feeding behavior, nutrient intake and performance of an herbivorous amphipod
Source: PeerJ. 2018 Nov 9;6:e5929. doi: 10.7717/peerj.5929 (PMC6231427; doi:10.7717/peerj.5929)
Supplement: Table S1 [file peerj-06-5929-s003.docx]

Table S1. t test for unequal variances comparing food mass change between grazed and ungrazed cups by trial in no-choice feeding experiment.

| Diet | t | *P* |
| --- | --- | --- |
| First trial |  |  |
| *Egregia* | 8.368 | < 0.001 |
| *Endarachne* | 5.075 | 0.002 |
| *Hormophysa* | 3.785 | 0.009 |
| *Padina* | 4.212 | 0.004 |
| *Sargassum* | 3.013 | 0.030 |
| *Turbinaria* | 2.831 | 0.024 |
| *Ulva* | 3.268 | 0.022 |
| Second trial |  |  |
| *Egregia* | 3.770 | 0.004 |
| *Endarachne* | 3.283 | 0.009 |
| *Hormophysa* | 0.970 | 0.347 |
| *Padina* | 2.795 | 0.021 |
| *Sargassum* | 2.956 | 0.016 |
| *Turbinaria* | 1.024 | 0.332 |
| *Ulva* | 3.345 | 0.009 |
| Third trial |  |  |
| *Egregia* | 3.359 | 0.010 |
| *Endarachne* | 1.404 | 0.197 |
| *Hormophysa* | 3.12 | 0.012 |
| *Padina* | 1.581 | 0.158 |
| *Sargassum* | 2.759 | 0.025 |
| *Turbinaria* | 0.267 | 0.795 |
| *Ulva* | 2.499 | 0.037 |
